# Probiotic extracellular vesicles reprogram macrophage immunometabolism: From gut crosstalk to host health

**DOI:** 10.1080/19490976.2026.2614115

**Published:** 2026-01-11

**Authors:** Yan Li, Yihong Liu, Xubiao Wei, Changfa Wang, Muhammad Zahoor Khan, Qingshan Ma

**Affiliations:** a College of Agriculture and Biology, Liaocheng University, Liaocheng, Shandong, People's Republic of China; b College of Animal Science and Technology, China Agricultural University, Beijing, People's Republic of China

**Keywords:** Probiotics, bacterial extracellular vesicles, immunometabolism, macrophage, metabolic pathways

## Abstract

Probiotic-derived extracellular vesicles (PEVs) are functional nanovesicles secreted by various microbiota. As a novel class of microbial signals, they encapsulate proteins, nucleic acids, lipids, and microbial-associated molecular patterns, emerging as potent modulators of communication between gut microbiota and host immune cells, such as macrophages. Macrophages, as a crucial component of the innate immune system, rely heavily on specific metabolic reprogramming to execute their immune functions effectively. Recent evidence demonstrates the pivotal role of macrophage immunometabolism in orchestrating inflammatory responses and regulating systemic metabolic health. This review provides the first comprehensive synthesis of current evidence linking PEVs to the function and metabolic reprogramming of macrophages. We first conducted a detailed exploration of the release rationale, biosynthesis, composition, uptake by macrophages, and biological activity of PEVs. Subsequently, we elucidated how these vesicles and their cargo influence macrophage polarization through several metabolic pathways, including glycolysis, oxidative phosphorylation (OXPHOS), fatty acid oxidation (FAO), and amino acid metabolism. We further explore the implications of macrophage immunometabolism in chronic inflammation and metabolic disorders, including inflammatory bowel disease (IBD), neurodegenerative diseases, and atherosclerosis. Additionally, emerging evidence indicates that PEVs may be influenced by various factors, which in turn can affect host immunity and metabolism. Finally, we briefly discuss the limitations and future challenges in this field. This review highlights new research targets concerning the impact of gut microbiota on host immunity and metabolism.

## Graphical abstract

**Figure:**
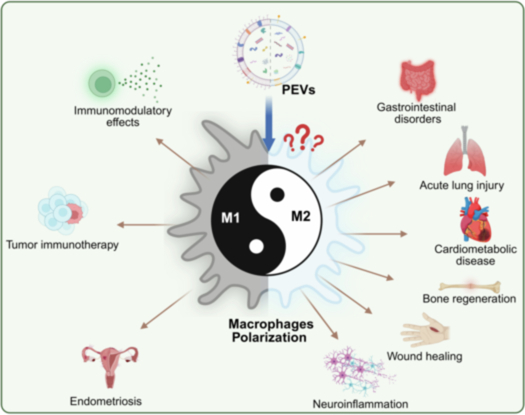

## 1 Introduction

Macrophages are critical components of the innate immune system, widely distributed across various organs, and play indispensable roles in host defense against external infections, maintaining internal homeostasis, and orchestrating immune regulation. Their remarkable plasticity enables dynamic phenotypic transitions in response to environmental cues, with metabolic reprogramming serving as a central determinant of functional polarization.^1‐3^ M1 macrophages are typically activated by lipopolysaccharides (LPS) or Th1-associated cytokines, such as interferon-gamma (IFN-*γ*), and are characterized by their pro-inflammatory properties, relying predominantly on aerobic glycolysis (the Warburg effect) with disrupted tricarboxylic acid (TCA) cycles that accumulate signaling metabolites including succinate and citrate.^4‐6^ In contrast, M2 macrophages are polarized by Th2-associated cytokines, including interleukin-4 (IL-4) and interleukin-10 (IL-10), exhibiting anti-inflammatory functions supported by oxidative phosphorylation (OXPHOS) and fatty acid oxidation (FAO), maintaining intact TCA cycle activity.^7‐9^ This metabolic-functional coupling has emerged as a promising therapeutic target, with immunometabolic dysregulation contributing to chronic inflammatory disorders including obesity, atherosclerosis, and inflammatory bowel disease.^10‐17^

Intestinal probiotics play a central role in regulating host metabolic and immune homeostasis, with macrophages serving as critical effector cells that bridge innate and adaptive immunity.^18^ Probiotic-derived extracellular vesicles (PEVs) are membrane-bound vesicles actively released by probiotic bacteria, ranging in size from 20 to 400 nm, which are capable of encapsulating bioactive cargos, including proteins, lipids, and small RNAs.^19^ Unlike their parental bacteria, PEVs circumvent viability constraints, traverse epithelial barriers, and deliver cargo directly to target immune cells with enhanced stability and reduced immunogenicity.^20‐22^ Increasing evidence indicates that PEVs act as potent immunoregulatory agents capable of modulating macrophage function through direct metabolic reprogramming, altering key pathways such as glycolysis, OXPHOS, FAO, and cholesterol efflux.^23‐25^

Given the central importance of macrophage immunometabolism in shaping host-microbiota interactions, inflammatory resolution, and systemic metabolic health, it is imperative to gain a detailed understanding of how PEVs influence this regulatory axis. Uncovering these mechanisms not only provides fundamental insights into host-microbe crosstalk but also opens new therapeutic avenues. Therefore, this review first address fundamental questions regarding the physiological rationale for the release, biosynthesis, molecular composition, uptake by macrophages and biological activity of PEVs, as well as their relevance mechanism such as cargo loading mechanisms and the molecular determinants distinguishing pro-inflammatory from anti-inflammatory outcomes. We then provide a focused mechanistic analysis of PEVs-macrophage interactions, emphasizing immunometabolic reprogramming pathways. Furthermore, we explore application strategies involving delivery systems and multi-omics based analysis to harness PEVs as therapeutic tools for disease management, highlighting their potential for next-generation interventions in immune-metabolic diseases. Throughout, we critically evaluate evidence quality, explicitly distinguish mechanistically demonstrated effects from speculation, and identify key knowledge gaps requiring experimental resolution.

## 2 Basic of PEVs

The PEVs are nanoscale, bilayered structures actively released by various probiotic taxa. These vesicles serve as evolutionarily conserved messengers in microbe-host communication. In the following sections, we examine the release rationale, biogenesis and structural characteristics of PEVs, as well as their interactions with host immune cells, thereby establishing a foundation for understanding their functional impact on macrophages.

### 2.1 Physiological rationale for bacterial EV release

Understanding why bacteria release extracellular vesicles is fundamental to interpreting their biological significance.^26^ Bacterial vesiculation represents an evolutionarily conserved process observed across diverse prokaryotic phyla, suggesting strong selective pressures maintaining this phenotype.^27^ Contemporary research has identified multiple, non-mutually exclusive functions for bacterial EV release that can be categorized into ecological drivers, evolutionary advantages, and mechanistic triggers.^28^

#### 2.1.1 Ecological drivers of vesiculation

In their native ecosystems, bacteria face intense competition for resources and must defend against bacteriophages, antimicrobial compounds, and host immune responses. EVs serve multiple ecological functions that enhance bacterial fitness.^29^ At the most fundamental level, EVs function as decoy targets for bacteriophages, providing membrane-bound structures that can absorb phage particles, thereby reducing productive infection of parent cells.^30^ Beyond this defensive role, EVs facilitate nutrient acquisition through the delivery of hydrolytic enzymes to distant substrates, enabling cooperative resource exploitation within bacterial communities.^30^ EVs also mediate inter-bacterial competition by delivering bacteriocins, toxins, and cell wall-degrading enzymes to competitor organisms, as demonstrated for *Pseudomonas aeruginosa* EVs carrying *β*-lactamases that protect neighboring cells.^31^ Of particular relevance to host-microbe interactions, EVs enable communication across the gut mucus layer, allowing bacteria to signal to distant host cells without requiring direct contact or translocation.

#### 2.1.2 Evolutionary roles of EV production

From an evolutionary perspective, vesiculation confers several fitness advantages that explain its conservation across bacterial lineages. EVs enable horizontal gene transfer by packaging and protecting DNA during transfer between cells, contributing to bacterial adaptation and antibiotic resistance dissemination.^32^^,^^33^ EVs facilitate biofilm formation and maintenance by delivering matrix components and signaling molecules.^34^ Perhaps most significantly for host-associated bacteria, EVs enable long-distance signaling to host cells, allowing commensal organisms to shape mucosal immunity without triggering the inflammatory responses associated with bacterial translocation.^35^ This “stealth communication” may represent a co-evolved strategy benefiting both host and microbe.^36^

#### 2.1.3 Intentional signaling versus stress-induced release

A critical unresolved question concerns whether EV release represents active, regulated secretion or a passive consequence of cellular stress. Current evidence supports both mechanisms operating in context-dependent fashions.^37^^,^^38^ In Gram-negative bacteria, bacterial extracellular vesicle (BEV) formation primarily involves two distinct pathways: membrane blebbing, which produces outer membrane vesicles (OMVs) through regulated budding, and explosive cell lysis, which generates outer-inner membrane vesicles (OIMVs) enriched with cytoplasmic contents.^37^^,^^39^

Active, intentional vesiculation is evidenced by the existence of genetic regulators controlling EV production rates. The VacJ/Yrb ABC (ATP-binding cassette) transport system represents a highly conserved mechanism for regulating OMV biogenesis in Gram-negative bacteria.^40^ This phospholipid transporter maintains lipid asymmetry in the outer membrane by retrograde trafficking of phospholipids from the outer to inner membrane. Deletion or repression of VacJ/Yrb genes leads to phospholipid accumulation in the outer leaflet of the outer membrane, causing asymmetric expansion that initiates membrane blebbing and OMV formation.^40^^,^^41^ Importantly, this regulatory mechanism is controlled by environmental signals, particularly iron availability, with iron limitation leading to ferric uptake regulator (Fur)-dependent downregulation of the VacJ/Yrb system and consequent hypervesiculation.^40^^,^^42^ The selective enrichment of specific cargo molecules in EVs relative to parent cell membranes provides additional evidence for regulated secretion, as proteomic analyzes have demonstrated that OMV protein composition differs dramatically depending on culture conditions and can be remodeled in response to environmental cues.^43^^,^^44^

Stress-induced vesiculation is supported by extensive observations that various environmental stressors significantly affect BEV production and cargo composition. Antibiotic exposure substantially enhances BEV secretion, cargo enrichment, and motility through complex molecular mechanisms.^44^ For example, polymyxin B exposure markedly increases OMV secretion by *Klebsiella pneumoniae*, with these vesicles competitively binding the antibiotic to protect bacteria.^45^ Similarly, sub-inhibitory concentrations of metronidazole and levofloxacin induce EV secretion through their DNA-targeting activity and ability to generate oxidative stress.^46^ Under oxidative stress conditions, *Staphylococcus aureus* exhibits significantly higher MV production, while *Helicobacter pylori* responds by secreting catalase-containing EVs that promote bacterial survival.^38^ Temperature fluctuations, nutrient limitation, and pH changes also modulate vesicle production and cargo composition, representing bacterial adaptation strategies to cope with environmental changes.^47^

Explosive cell lysis represents a distinct stress-induced mechanism contributing substantially to EV pools under certain conditions. Consistently, a study demonstrated that explosive lysis of a sub-population of cells accounts for the liberation of cytosolic content in *Pseudomonas aeruginosa* biofilms.^30^ Super-resolution microscopy revealed that shattered membrane fragments produced by exploding cells rapidly curl and self-anneal to form membrane vesicles that capture extracellular DNA and other cellular components. This process is mediated by a prophage endolysin encoded within the R- and F-pyocin gene cluster, and endolysin-deficient mutants are defective in MV production and biofilm development.^30^^,^^37^ Exposure to stress conditions, including antibiotics and DNA-damaging agents, stimulates expression of the gene encoding the endolysin and induces explosive cell lysis, demonstrating that the bacterial SOS response can trigger this lysis-dependent vesiculation pathway.^30^

The relative contributions of intentional versus incidental vesiculation likely vary by species, growth phase, and environmental conditions.^37^^,^^47^ Multiple biogenesis pathways may operate simultaneously, reflecting the complexity and adaptability of bacterial envelope dynamics, with stress conditions such as nutrient limitation, oxidative stress, or antibiotic exposure preferentially activating specific pathways and thereby altering EV composition and functionality.^47^ For probiotic-derived EVs specifically, studies suggest that both mechanisms contribute to host-microbe communication. Baseline constitutive release provides ongoing immunomodulatory signaling, with probiotic EVs activating host immune cells through Toll-like receptor (TLR2 and TLR4) signaling pathways and inducing production of both pro- and anti-inflammatory cytokines.^35^ Meanwhile, stress-induced hypervesiculation may represent an adaptive response to hostile gut environments, as demonstrated by *Lactobacillus*-derived EVs that orchestrate intestinal barrier maturation through macrophage-epithelial crosstalk and suppress NLRP3 inflammasome activation.^39^^,^^48^

#### 2.1.4 *In vivo* versus *In vitro* EV behavior

An important caveat for interpreting the PEV literature concerns potential differences between EV behavior in controlled laboratory conditions versus complex in vivo environments. In vitro studies typically employ EVs isolated from bacteria grown in rich media under optimal conditions, whereas in vivo, probiotic bacteria experience fluctuating nutrient availability, bile acid exposure, pH variation, and competitive interactions with other microorganisms.^49^^,^^50^ These environmental factors influence EV production rates (bile acids increase EV release up to 100-fold from *L. johnsonii*), cargo composition (stress conditions alter protein and RNA profiles), membrane lipid composition (affecting fusion capacity and stability), and surface modifications (affecting immune recognition and tropism).^50‐52^ Furthermore, in vivo biodistribution studies remain limited, making it uncertain whether EVs produced in the gut lumen reach systemic circulation in quantities sufficient for extraintestinal effects.^50^^,^^53^^,^^54^ Most therapeutic claims derive from studies administering purified EVs at supraphysiological doses via routes (intraperitoneal, intravenous) that bypass normal gut processing.^55^ Whether endogenously produced EVs achieve comparable tissue concentrations remains to be determined, particularly given that EVs exhibit natural biodistribution concentrated primarily in the liver and spleen with short plasma half-life, presenting significant challenges to clinical translation.^56^^,^^57^ This in vitro-in vivo disconnect represents a significant knowledge gap that must be addressed before therapeutic translation.^40,58‐60^

### 2.2 Biogenesis of PEVs

**Figure 1.**
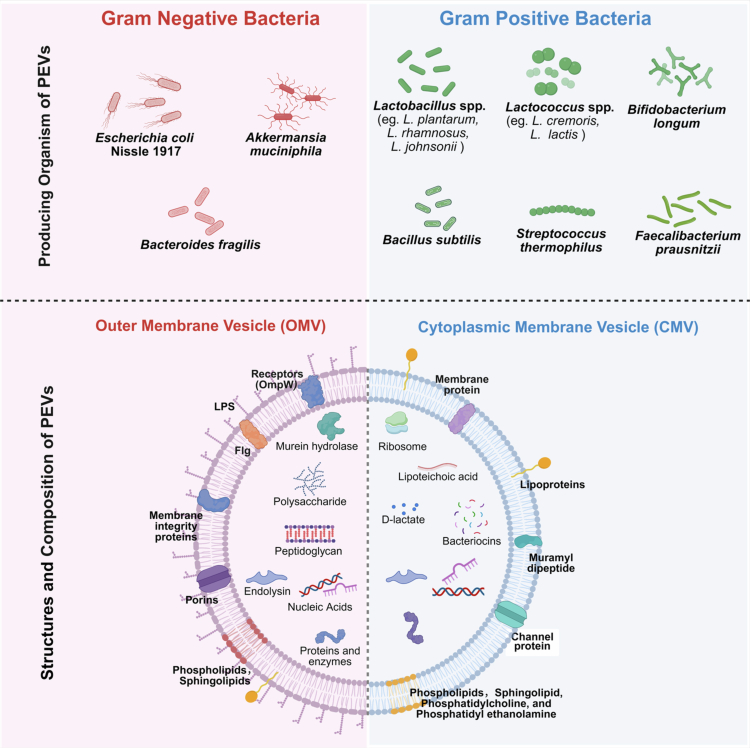
Structures and composition of Probiotics-derived extracellular vesicles (PEVs) from Gram-positive and Gram-negative bacteria. The cargos in PEVs from Gram-negative and Gram-positive bacteria differ slightly. The figure was created with https://app.biorender.com/.

PEV biogenesis is fundamentally shaped by the cell envelope architecture of the parent bacterium.^61^ Gram-negative probiotic vesicles are generated through multiple routes, whereby outer membrane blebbing produces OMVs, while explosive cell lysis yields outer-inner membrane vesicles (OIMVs) and explosive outer membrane vesicles (EOMVs), each carrying distinct cargo including DNA, RNA, and metabolic enzymes.^37^^,^^39^^,^^62^^,^^63^ Mechanistically, OMV formation in Gram-negative bacteria is driven by the accumulation of misfolded proteins in the periplasm, reduced crosslinks between the outer membrane and peptidoglycan layer, and insertion of curvature-inducing molecules such as LPS and phospholipids into the outer leaflet.^40^^,^^62^ Specific genetic regulators, including the vacJ/yrb ABC transport system, modulate vesiculation rates by controlling phospholipid trafficking between inner and outer membranes.^40^ Time-lapse imaging of *Escherichia coli* has demonstrated OMV production through both membrane blebbing and explosive lysis,^63^ while EV formation in *Stenotrophomonas maltophilia* is mediated by cryptic tailocin endolysin and regulated by DNA-damaging agents.^64^ In contrast, Gram-positive bacteria produce cytoplasmic membrane vesicles (CMVs) that traverse the thick peptidoglycan barrier via localized wall remodeling, prophage-encoded holin-lysin systems, or endolysin-mediated cell wall rupture.^65‐68^ The peptidoglycan layer, which ranges from 20–80 nm in Gram-positive organisms,^69^ presents a significant physical barrier to vesicle release.^70^^,^^71^ Current models propose that localized enzymatic degradation by autolysins creates transient openings,^66^^,^^72^ while turgor pressure facilitates membrane protrusion through these gaps.^71^ Studies in *Bacillus subtilis* revealed that phage-encoded endolysin creates pores allowing cytoplasmic membrane material to protrude and release as EVs,^66^ while STORM (Stochastic Optical Reconstruction Microscopy) and scanning electron microscopy identified membrane blebbing and explosive lysis as primary biogenesis pathways in *Staphylococcus aureus.*^63^ In *Lactococcus lactis*, EV formation is stimulated by prophage-encoded holin-lysin systems through gradual plasma membrane extrusion without immediate lysis,^67^ and *Streptococcus thermophilus* produces EVs through a prophage-encoded lysozyme-lycocin system.^68^ CMVs contain cytoplasmic cargo analogous to EOMVs and OIMVs, playing crucial roles in host immune interactions,^73^ with structures and composition shown in Figure 1. Environmental regulation significantly influences PEV biogenesis, as the rate, protein content, and yield respond to environmental stressors including nutrient deprivation, temperature shifts, and oxidative stress,^26^ and PEV biogenesis is both strain-dependent and condition-specific,^48^^,^^49^ suggesting dynamic regulation by host diet, microbial competition, or environmental factors.

### 2.3 Cargo selection and molecular composition

PEVs typically range from 20–400 nm in diameter with bilayer membranes enriched with lipids, proteins, and selectively packaged nucleic acids.^28^^,^^37^^,^^49^ Cargo loading is an active, selective process driven by electrostatic and lipid-protein interactions,^62^ underpinning PEVs' ability to modulate host immune and metabolic pathways,^21^^,^^22^ with key cargo categories summarized in Table 1. The mechanisms governing cargo selection remain incompletely understood but involve several processes operating in parallel.^37^^,^^74^ For proteins, selective enrichment correlates with membrane association, charge characteristics, and specific targeting sequences. Proteins enriched in EVs relative to parent cells often contain lipid-binding domains, oligomerization motifs, or post-translational modifications (particularly acylation and prenylation) that promote membrane association.^37^ For RNA cargo, selective packaging appears mediated by specific sequence motifs recognized by RNA-binding proteins, secondary structures promoting membrane association, and co-packaging with protein complexes. Studies in *Salmonella* demonstrate that certain sRNAs are enriched 10-100 fold in EVs compared to cellular RNA pools, indicating active sorting rather than passive encapsulation.^75^ Similarly, studies in *Bacteroides fragilis* have shown that OMVs are enriched in DNA, RNA, and protein cargo compared to their parent bacteria, with OMVs capable of transporting bacterial RNA directly into host epithelial cells.^76^ For lipids, asymmetric distribution between inner and outer membrane leaflets, coupled with lipid raft-like microdomains, creates local environments favoring vesicle budding. Sphingolipid-enriched microdomains appear particularly important for cargo sorting in *Bacteroides* species, with lipidomic analyzes revealing that sphingolipids constitute more than 50% of total lipid content in *Bacteroides thetaiotaomicron* OMVs.^77^

**Table 1.** Composition of probiotic extracellular vesicles (PEVs) organized by mechanistic category.

Mechanistic category	Cargo class	Representative cargo	Primary biological effects	Key references
***A. Direct Immunomodulation (Cargo → PRR → Immune Signaling)***				
TLR Activation	Proteins	Flagellin (FlgA, FlgE, FlgK); OmpA, OmpC; Lipoproteins (Lp19180, Pal)	Innate immune activation; NF-κB signaling; TLR2/4-mediated cytokine production	[78‐82]
TLR Activation	Lipids	LPS; Lipoteichoic acid; Lipooligosaccharide; Sphingolipids	TLR2/4-mediated immune responses; Anti-inflammatory signaling	[83‐88]
NOD Activation	Polysaccharides	Peptidoglycan; *β*-D-glucan; Muramyl dipeptide	NOD2-mediated signaling; Innate immune maintenance	[89‐92]
***B. Direct Gene Regulation (Cargo → Host Gene/Protein Modulation)***				
miRNA-mediated	Nucleic Acids	let-7i; miR-146b; miR-5119; miR-4239; sRNAs	Cytokine gene regulation; PD-L1 modulation; IL-17a targeting	[93‐97]
Protein-mediated	Proteins	P40/P75; Amuc_1100; Amuc_1434; GAPDH; Protein P9	GLP-1/STAT3 signaling; PD-L1 expression; Epithelial protection	[49,93,98‐101]
***C. Direct Antimicrobial Activity (Cargo → Pathogen Inhibition)***				
Bacteriocins	Proteins	Nisin Z; Lactacin B; Rhs repeat proteins; Bacteriolytic endopeptidases	Direct pathogen killing; Biofilm inhibition; Inter-bacterial competition	[102‐106]
Enzymes	Proteins	Murein hydrolases (mltA, mltC); Lysozyme; Endolysins (Sdp)	Cell wall degradation; Antiviral activity	[83,107‐109]
***D. Metabolic Regulation (Cargo → Host Metabolism)***				
Metabolic Enzymes	Proteins	GAPDH; Enolase; LDH; Glucokinase; Phosphofructokinase	Glucose-lipid metabolism; Calcium signaling; Thermogenesis	[68,93,110,111]
Bioactive Lipids	Lipids	Phospholipids (PA, PC, PE, PG); Glycerolipids	Cell signaling; Energy metabolism; Membrane transport	[93,112‐114]
***E. Structural & Delivery Functions***				
Membrane Components	Proteins/Lipids	OmpA; BamB; LpoA; Glycerophospholipids	Cargo protection; Host cell adhesion; Delivery to target tissues	[78,79,114,115]

Note: Representative examples consolidated by mechanism of action. Direct effects indicate cargo acting directly on host pathways; structural components enable cargo delivery. Abbreviations: PRR, pattern recognition receptor; TLR, toll-like receptor; GLP-1, glucagon-like peptide-1; STAT3, signal transducer and activator of transcription 3;PD-L1, programmed cell death ligand 1; NOD, nucleotide-binding oligomerization domain; GAPDH, glyceraldehyde-3-phosphate dehydrogenase; LDH, lactate dehydrogenase.

PEVs carry diverse nucleic acids including DNA fragments, mRNA, and miRNAs that modulate gene expression and host function.^116^ *Akkermansia muciniphila* vesicles contain let-7i miRNAs modulating pro-inflammatory gene expression,^93^ while *Faecalibacterium prausnitzii* vesicles harbor various miRNAs (miR-4270, miR-6787, miR-6602), lncRNAs, and snoRNAs.^117^ *Bacteroides fragilis* vesicles contain miR-5119-like sRNAs that suppress PD-L1 expression, attenuate NET formation, and promote intestinal stem cell proliferation.^94^ Additionally, miR-146b from *Lactococcus lactis* promotes M2 macrophage polarization,^95^ while sRNAs from *Lactiplantibacillus plantarum* reduce IL-8 production,^96^ highlighting vesicle-carried RNAs as emerging postbiotic regulators with therapeutic potential.^49^^,^^118^ The functional consequences of RNA delivery depend on RNA stability following host cell uptake. Studies using fluorescently labeled RNAs demonstrate that EV-delivered bacterial sRNAs can persist for 12-24 hours within host cells, sufficient time to modulate target gene expression.^119^^,^^120^

Proteins represent functionally diverse PEV components with profiles reflecting parent bacterium architecture. In Gram-negative bacteria, *E. coli* Nissle 1917 OMVs contain structural proteins (OmpA, OmpC, OmpX) facilitating adhesion and immune interactions,^78^^,^^79^^,^^121^ alongside murein hydrolases with antimicrobial activity,^83^ while *A. muciniphila* OMVs contain Amuc_1434 enhancing CD8+ T-cell immunity and Amuc_2172 promoting M1 macrophage polarization.^98^^,^^122^^,^^123^ In Gram-positive bacteria, CMVs typically harbor cytosolic proteins with immunomodulatory or antimicrobial properties, including P40 and P75 from *Lacticaseibacillus paracasei* with anti-apoptotic and barrier-protective effects,^99^^,^^124^ GAPDH and lipopeptide ligands from *L. plantarum* modulating TLR2-NF-κB signaling,^80^^,^^100^^,^^125^ and bacteriocins such as nisin Z from *Lactococcus lactis* and lactacin B from *Lactobacillus acidophilus.*^102^^,^^103^ Other PEVs contain immune-related proteins participating in colonization,^126^ metabolism,^117^ and immune regulation,^81^^,^^104^ and proteomic profiling has identified metabolic enzymes influencing nutrient processing and host responses,^110^^,^^111^^,^^127^ though most functional insights require in vivo validation.

Lipids provide structural foundation while exerting immunomodulatory functions. Gram-negative PEV lipids typically contain LPS stimulating innate immunity,^83^^,^^93^ and *A. muciniphila* OMVs contain sphingolipids and lipooligosaccharides with TLR signaling activity underlying anti-inflammatory effects.^84^^,^^85^ *Alistipes timonensis* vesicles carry sulfonolipids suppressing pro-inflammatory cytokines in macrophages,^128^ while sphingolipids from *Bacteroides thetaiotaomicron* OMVs trigger IL-10 production via the mevalonate pathway.^24^ Gram-positive PEV lipids are characterized by lipoteichoic acids (LTA), with LTA from *L. plantarum* and *L. rhamnosus* exhibiting immunomodulatory functions analogous to LPS,^86^^,^^87^ and lipidomic profiling has identified diverse phospholipids (PA, PC, PE, PG, PI) that stabilize membranes while engaging host signaling.^112^^,^^113^

PEVs also encapsulate polysaccharides and small-molecule metabolites, including peptidoglycan fragments from EcN and *A. muciniphila* OMVs that activate innate immunity,^89^^,^^93^ polysaccharide A from *B. fragilis* OMVs promoting immune regulation,^90^ and yeast-derived vesicles containing *β*-glucans that activate macrophages and dendritic cells via TLR2.^91^ Metabolomic studies demonstrate PEVs transport bioactive metabolites including indoles, amino acids, and short-chain organic acids,^117^^,^^129^ with *F. prausnitzii* EVs containing inosine and succinic acid influencing metabolic networks,^117^ and *Lactobacillus crispatus*-derived EVs carrying D-lactate promoting M2 macrophage polarization and wound healing.^130^

The mechanisms by which PEV cargo reaches functional compartments within host cells are increasingly understood.^131^^,^^132^ Following receptor-mediated uptake, EVs traffic through the endosomal system, with cargo fate determined by vesicle composition and cell type. Several non-mutually exclusive delivery routes have been identified. Direct membrane fusion at the plasma membrane or within endosomes represents one major pathway, releasing luminal cargo directly into the cytoplasm; this route is facilitated by low pH in late endosomes and specific lipid compositions.^133^ Alternatively, endosomal escape mediated by pore-forming proteins or LPS-induced membrane destabilization allows protein and RNA cargo to access cytoplasmic targets.^132^ In addition, endosomal rupture, particularly following NLRP3 inflammasome activation, releases EV contents into the cytosol while simultaneously triggering inflammatory signaling.^134^^,^^135^ Furthermore, transcytosis across epithelial barriers allows intact EVs to reach the lamina propria where they encounter immune cells. For macrophages specifically, pattern recognition receptors (TLRs, NOD1/2, scavenger receptors) serve as primary recognition and uptake mediators.^136^ TLR2 and TLR4 engagement by lipoproteins and LPS respectively triggers receptor-mediated endocytosis while simultaneously initiating signaling cascades.^132^ This coupling of uptake and activation ensures that cargo delivery occurs in the context of appropriate immune priming. Cargo loading involves passive mechanisms such as random encapsulation during blebbing, and active processes including selective sorting through electrostatic interactions, protein-protein interactions, and lipid raft-mediated partitioning. PEVs deliver cargo through receptor-mediated endocytosis, macropinocytosis, direct membrane fusion, and lipid raft-mediated uptake.^137^ For macrophages, pattern recognition receptors (TLR2, TLR4, NOD1, NOD2) serve as primary sensors, triggering downstream signaling through MyD88-dependent and -independent pathways, and these interactions determine whether PEVs elicit pro-inflammatory responses via NF-κB and MAPK activation or anti-inflammatory responses via STAT6 and PPARγ signaling.^138^ The relative contributions of different cargo classes to specific immunometabolic outcomes remain to be systematically delineated.

### 2.4 Uptake mechanisms by host cells

Mounting evidence indicates that PEVs can efficiently cross mucosal barriers and preferentially accumulate in immune compartments, with macrophages identified as major target cells. This selective targeting provides a mechanistic foundation for understanding how PEVs mediate their immunomodulatory effects.

#### 2.4.1 Preferential uptake by macrophages

Fluorescence imaging studies have demonstrated that PEVs exhibit remarkable tropism for macrophages in various tissue contexts. Vesicles derived from *Faecalibacterium prausnitzii* can traverse the intestinal epithelium and localize to the lamina propria, where they co-localize with F4/80⁺ macrophages rather than CD3⁺ T cells, with over 90% of colonic macrophages internalizing DiI-labeled *F. prausnitzii* EVs within 12 hours, suggesting preferential targeting of macrophages to mediate anti-inflammatory effects in colitis models.^25^ Similar observations have been reported for *E. coli* and *Lactobacillus*-derived EVs, which induce alterations in macrophage polarization and cytokine production following uptake.^139^^,^^140^ Beyond the gut, certain PEVs exhibit preferential uptake by macrophage subsets in inflamed tissues, with vesicles derived from *Lactobacillus rhamnosus* accumulating in plaque-resident foamy macrophages in atherosclerotic models, indicating potential passive targeting through enhanced lesion permeability.^23^

#### 2.4.2 Cellular entry mechanisms

Mechanistically, PEVs enter host cells through multiple pathways including endocytosis, direct membrane fusion, lipid raft-mediated internalization, and paracellular translocation across epithelial barriers,^141^ with the specific route determined by vesicle size, surface composition, and recipient cell activation state. Endocytic pathways represent the predominant route of PEVs internalization, with vesicles from *Bacteroides thetaiotaomicron* internalized into epithelial cells via dynamin-dependent endocytosis, subsequently trafficked to LAMP-1⁺ endo-lysosomal compartments and redistributed to perinuclear regions,^142^ and comparable routes reported for bifidobacterial EVs^143^ and *Lactiplantibacillus plantarum* EVs through clathrin-mediated endocytosis.^144^ Size-dependent mechanisms influence macrophage uptake, with small vesicles (20-100 nm) typically employing caveolin- or clathrin-dependent endocytosis while larger vesicles (90-450 nm) preferentially utilize macropinocytosis.^145^ Specific receptor-ligand pairs mediating macrophage recognition of PEVs include: TLR2 recognizing lipoproteins, LTA, and peptidoglycan fragments, leading to MyD88-IRAK-TRAF6-NF-κB signaling; TLR4 recognizing LPS (in Gram-negative EVs), activating both MyD88-dependent and TRIF-dependent pathways^146^; NOD1 and NOD2 recognizing muropeptides following endosomal processing, activating RIPK2-NF-κB signaling^147^; scavenger receptors (SR-A, CD36, LOX-1) mediating lipid-dependent uptake; and complement receptors recognizing opsonized vesicles. The specific receptor engaged depends on EV composition and significantly influences downstream functional outcomes.^148^ For example, TLR2-mediated uptake of L. rhamnosus EVs promotes anti-inflammatory IL-10 production, while TLR4-mediated uptake of *E. coli* EVs triggers pro-inflammatory TNF-*α* release.^80^^,^^148^ Furthermore, receptor-ligand interactions confer selectivity, as *E. coli*-derived OMVs functionalized on gold nanocages are preferentially internalized by CD64⁺ and CD14⁺ M1 macrophages, whereas *L. rhamnosus*-derived EVs enhance bacterial phagocytosis via FPR1/2 signaling.^140^^,^^149^

#### 2.4.3 Post-internalization signaling and macrophage fate

Beyond uptake routes, downstream molecular mechanisms dictate macrophage functional fate following PEVs internalization, determining whether vesicle uptake results in pro-inflammatory activation, anti-inflammatory polarization, or cell death. *Staphylococcus aureus* EVs are internalized via dynamin-dependent endocytosis, triggering TLR2-mediated NLRP3 inflammasome activation leading to caspase-1 activation and pyroptotic cell death accompanied by IL-1β and IL-18 release,^150^ and similarly *Klebsiella pneumoniae* EVs promote macrophage pyroptosis through uptake via the lectin-like oxidized LDL receptor.^151^ In contrast, probiotic-derived vesicles induce anti-inflammatory polarization through distinct mechanisms: EVs from *Streptococcus pneumoniae* induce NF-κB activation in a dose-dependent manner promoting M2 macrophage polarization,^152^ while *Lactobacillus reuteri* EVs localize to mitochondria where they reduce oxidative stress through 3-hydroxypropionaldehyde, thereby favoring M2 polarization.^153^ The structural features of vesicles, including surface glycans and proteins, influence both efficiency and immunological outcomes of macrophage internalization, as evidenced by differential cytokine responses to fungal EVs with distinct surface coats.^154^

Collectively, the phenomenon of bacteria vesiculation fundamentally reflects the regulatory properties of microorganisms in ecological adaptation and host interaction. By selectively releasing vesicles, this mechanism facilitates the signal transmission and the regulation of immune homeostasis, which holds significant physiological importance. PEVs exploit this physiological mechanism, utilizing their nanoscale structure and specific cargo-carrying capacity to traverse host barriers, target immune cells, and modulate signaling pathways. This positions them as novel “cell-free effectors” distinct from their parent bacteria. By activating receptors such as TLR2 and NOD1, along with key signaling axes like NF-κB and STAT6, PEVs reshape the functional and metabolic states of immune cells, particularly macrophages. These findings provide a mechanistic basis for the impact PEVs mucosal homeostasis and systemic immune remodeling, conceptually linking the universal biological properties of PEVs to the macrophage immune metabolic regulation discussed in subsequent sections.

## 3 Macrophage immunometabolic reprogramming mediated by PEVs

### 3.1 Overview of macrophage function and plasticity

Macrophages exhibit a spectrum of activation states with extraordinary functional plasticity in response to diverse environmental stimuli, including pathogen-associated molecular patterns (PAMPs), damage-associated molecular patterns (DAMPs), cytokines, and tissue-specific microenvironmental cues.^6^^,^^9^^,^^155^ This adaptability enables macrophages to dynamically modulate their functional programs to meet the evolving demands of their local tissue environment.

Classical M1 activation occurs through TLR engagement with PAMPs such as lipopolysaccharide (LPS) or through interferon-*γ* (IFN-*γ*) stimulation via the JAK-STAT1 pathway.^156^ M1 macrophages are characterized by enhanced expression of inducible nitric oxide synthase (iNOS), cyclooxygenase-2 (COX-2), production of reactive oxygen species (ROS) through NADPH oxidase, and robust secretion of pro-inflammatory cytokines including TNF-*α*, IL-1β, IL-6, IL-12, and IL-23.^157^^,^^158^ These macrophages exhibit enhanced microbicidal capacity, present antigens via upregulated MHC class II and co-stimulatory molecules (CD80, CD86), and drive Th1 and Th17 adaptive immune responses.

In contrast, alternative M2 activation encompasses multiple polarization states (M2a, M2b, M2c, M2d) with distinct triggering stimuli and functional characteristics.^1^ M2a macrophages are induced by IL-4 and IL-13 through IL-4Rα activation of JAK1/JAK3-STAT6 signaling, driving expression of arginase-1 (Arg1), mannose receptor (CD206), IL-10, and TGF-*β*, thereby supporting tissue repair and anti-inflammatory functions.^159^ M2b macrophages arise from combined TLR and immune complex stimulation, exhibiting immunoregulatory properties. M2c macrophages, induced by IL-10, TGF-*β*, or glucocorticoids, specialize in immune suppression and tissue remodeling. The functional versatility of M2 macrophages positions them as critical regulators in wound healing, parasite immunity, and tissue homeostasis.

### 3.2 Metabolic reprogramming in macrophage polarization

Macrophages are highly reliant on specific metabolic reprogramming to adapt to their environment during polarization and the execution of immune functions.^1^^,^^160^^,^^161^ The metabolic pathways include four categories: carbohydrate metabolism, lipid metabolism, amino acid metabolism, and nucleotide metabolism.^162^^,^^163^ In pro-inflammatory M1 activation, macrophages undergo rapid metabolic transformation prioritizing glycolysis—the Warburg effect—through coordinated upregulation of glucose transporter 1 (GLUT1), hexokinase-2 (HK2), PFKFB3, PKM2, and LDHA.^5^ Concomitantly, the TCA cycle becomes disrupted, leading to accumulation of succinate and citrate that function as signaling molecules amplifying inflammatory responses.^164^ Succinate stabilizes HIF-1α through competitive inhibition of prolyl hydroxylase enzymes, reinforcing glycolytic programming and pro-inflammatory gene expression.^165^^,^^166^ Similarly, citrate accumulation supports fatty acid synthesis for membrane expansion and inflammatory lipid mediator production.^167^ Additionally, under LPS and IFNγ stimulation, macrophage remodel purine and pyrimidine metabolism through the regulatory factor NO, thereby influencing host defense function.^163^

In marked contrast, M2 macrophages rely predominantly on oxidative phosphorylation (OXPHOS) and fatty acid oxidation (FAO), maintaining intact and highly active TCA cycles.^7^ FAO becomes the predominant fuel source through upregulation of CPT1A and *β*-oxidation enzymes,^168^ while intact TCA cycle function ensures adequate *α*-ketoglutarate availability for biosynthetic pathways involved in tissue repair.^169^ Additionally, M2 macrophages promote cholesterol efflux through LXR target genes including ABCA1 and ABCG1, preventing foam cell formation and supporting anti-inflammatory functions.^10^ The pentose phosphate pathway serves critical but distinct functions in both activation states: generating NADPH for NADPH oxidase-mediated ROS production in M1 cells while supporting antioxidant defense in M2 macrophages.^170^^,^^171^ This dual functionality exemplifies how identical metabolic pathways can be differentially utilized to support opposing immunological outcomes depending on the polarization state and functional demands of the macrophage.

### 3.3 Regulatory networks governing metabolic reprogramming

**Figure 2.**
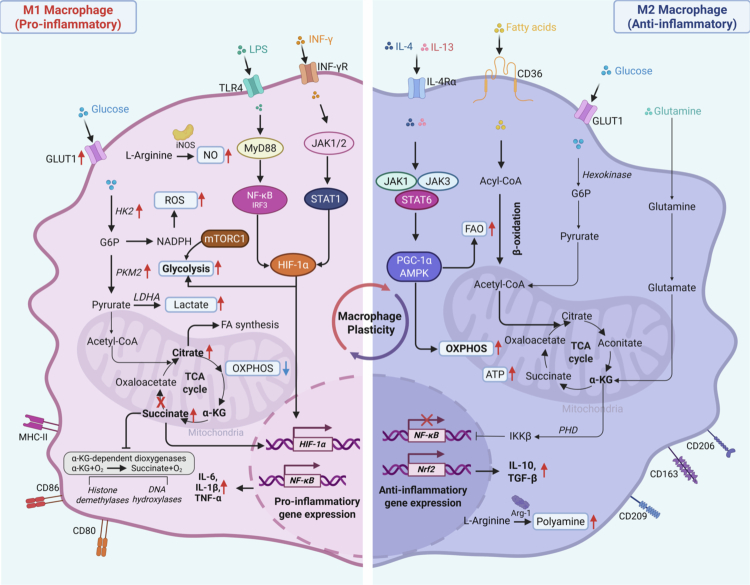
Overview of the regulatory pathways governing metabolic reprogramming in macrophages. Stimulated by LPS and IFN-*γ*, activating the MyD88→NF-κB/IRF3/HIF-1α and JAK-STAT1/HIF-1α pathways shifts metabolic activity primarily toward aerobic glycolysis (Warburg effect), promoting glucose conversion to pyruvate. Disruption of the TCA cycle leads to succinate and citrate accumulation, inhibiting OXPHOS. Succinate accumulation, competitively inhibits *α*-ketoglutarate-dependent dioxygenases, including both histone demethylases and DNA hydroxylases. L-Arginine is metabolized via the inducible nitric oxide synthase (iNOS) pathway to produce nitric oxide (NO), polarizing macrophages toward a pro-inflammatory phenotype (M1) and releasing pro-inflammatory cytokines. Under the influence of IL-4 and IL-13, activation of JAK1/JAK3-STAT6/PGC-1α and AMPK pathways, with metabolic activity primarily focused on FAO and OXPHOS. The metabolic intermediate *α*-KG inhibits the NF-κB signaling pathway through PHD enzyme activity, leading to the polarization of macrophages toward an anti-inflammatory phenotype (M2) and the release of anti-inflammatory cytokine. Center circular arrow indicating macrophages can transition between states. GLUT1, glucose transporter 1; TLR4, Toll-like receptor 4; IFN-*γ*, Interferon-γ; HK2, hexokinase 2; G6P, glucose-6-phosphate; PKM2, Pyruvate kinase M2; LDHA, Lactate dehydrogenase; NADPH, Nicotinamide adenine dinucleotide phosphate hydrogen; MyD88, Myeloid differentiation primary response protein 88; JAK, Janus kinase; NF-κB, Nuclear factor kappa-B; IRF3, Interferon regulatory factor 3; STAT1, Signal transducer and activator of transcription 1; HIF-1α, Hypoxia Inducible Factor 1 Subunit Alpha; OXPHOS, oxidative phosphorylation; *α*-KG, *α*-Ketoglutaric acid; PGC-1α, Peroxisome proliferator-activated receptor-gamma coactivator-1alpha; AMPK, Adenosine 5‘-monophosphate (AMP)-activated protein kinase; FAO, fatty acid oxidation; PHD, prolyl hydroxylase; LPS, Lipopolysaccharides; FA, fatty acid; Nrf2, Nuclear factor erythroid 2-related factor 2; IL-4, interleukin-4; IL-6, Interleukin-6; IL-1β, Interleukin-1β; TGF-*β*, Transforming growth factor-β; IKKβ, Inhibitor of kappa B kinase. The figure was created with https://app.biorender.com/.

Metabolic reprogramming in macrophages operates through hierarchically organized regulatory networks that integrate transcriptional control, post-translational modifications, and epigenetic remodeling (Figure 2). HIF-1α functions as the master regulator of M1 glycolytic metabolism, directly transactivating genes encoding glucose transporters and glycolytic enzymes while promoting PDK1 expression to shunt pyruvate away from mitochondrial oxidation.^166^ NF-κB coordinates inflammatory gene expression with metabolic reprogramming, creating feed-forward loops that sustain M1 activation.^172^ In contrast, PGC-1α/PPARγ drives the oxidative metabolic program characteristic of M2 macrophages through coordinated induction of mitochondrial biogenesis and FAO machinery.^8^^,^^173^ These regulatory nodes represent key targets through which PEVs may exert immunometabolic effects on macrophages.

### 3.4 PEV-mediated modulation of macrophage phenotype

PEVs function as precision modulators of macrophage polarization, shifting immune balance toward tissue-reparative phenotypes. Substantial evidence demonstrates that PEVs induce M2 polarization, enhancing IL-10 and TGF-*β* release while suppressing pro-inflammatory responses.^23,174‐177^ The molecular determinants distinguishing pro-inflammatory from anti-inflammatory outcomes depend on several factors. Cargo composition plays a critical role: EVs enriched in LPS/LTA activate TLR4/TLR2-NF-κB signaling promoting M1 polarization, while EVs enriched in sphingolipids or specific miRNAs (e.g., miR-146b) activate STAT6/PPARγ signaling favoring M2 polarization.^178^^,^^179^ Uptake efficiency and route also influence outcomes: rapid, high-dose uptake through scavenger receptors tends to trigger inflammatory responses, while gradual uptake through specific integrins promotes tolerogenic outcomes.^180^ Host cell baseline state is another important determinant, as pre-existing inflammatory priming sensitizes macrophages to pro-inflammatory EV effects, while homeostatic conditions favor anti-inflammatory responses.^180^ Finally, the tissue microenvironment modulates EV-macrophage interactions, with hypoxic, acidic, or metabolically stressed environments significantly altering both EV release and uptake dynamics.^178^ Table 2 summarizes specific PEV cargo components and their associated immunomodulatory effects.

**Table 2.** PEV-mediated macrophage polarization: direct vs indirect mechanisms.

Effect pathway	Probiotic strain(s)	Disease model	Macrophage shift	Key outcomes	Molecular mechanism	References
***DIRECT EFFECTS: PEV Cargo → PRR/Receptor → Signaling Pathway → Macrophage Polarization***						
TLR2-dependent	*P. pentosaceus*; *S. cerevisiae*; *L. murinus*	Colitis; Inflammation; Gut disease	M1→M2	↑IL-10, Arg-1, PD-L1; ↓Pro-inflammatory cytokines; Wound healing	TLR2 activation → NF-κB modulation; LTA recognition	[181,182]
TLR4/NF-κB	*L. gasseri*; *L. reuteri*	Intestinal inflammation	M1→M2	↓IL-6, TNF-*α*, IL-1β; ↓ROS/mitochondrial superoxide	TLR4/NF-κB pathway inhibition; MAPK suppression	[183,184]
NLRP3 Inflammasome	*L. plantarum*; *L. johnsoni*i; *E. rectale; L. amylovorus*	Colitis; Diarrhea; Neonatal gut	M1→M2	↑Tight junctions (ZO-1, Occludin); ↓IL-1β, IL-18; Barrier restoration	NLRP3-Caspase-1-ASC inhibition; NF-κBIA upregulation	[100,176,185,186]
miRNA Cargo	*L. lactis* (miR-146b); *C. butyricum* (miR-199a-3p); *L. plantarum* (cbn-let-7)	Colitis; IBD; Acute lung injury	M1→M2	Anti-inflammatory cytokine induction; Anti-ferroptosis; Barrier protection	miR-146b→M2 activation; miR-199a-3p→map3k4→MAPK/NF-κB; cbn-let-7→Acsl4	[95,187,188]
Metabolic Reprogramming	*F. prausnitzii*; *L. rhamnosus*	Chronic colitis; Atherosclerosis	M1→M2	↓Glycolysis; ↓Cholesterol efflux; Anti-fibrotic effects	PPARγ modulation; NR1H3→ABCA1 lipid efflux; mTORC1 axis	[23,189]
STING Pathway	*B. fragilis*	Vascular calcification; Diabetes	→M2 (context)	Sema7a induction; Serpine1 upregulation	STING-Sgpl1-Sema7a; STING-Mef2d-Trib1 signaling	[174,190]
***INDIRECT EFFECTS: PEVs → Microbiota Modulation → Metabolites → Macrophage Polarization***						
SCFA-mediated	*L. plantarum* (fucoxanthin); *C. butyricum*; *B. longum*	Colitis; IBD	M1→M2	↑Butyrate/SCFA; Gut microbiota remodeling; Dysbiosis correction	Microbiota reshaping → ↑SCFA production → HDAC inhibition → M2 polarization	[175,188,191,192
Microbiota-Immune Axis	*E. coli* Nissle 1917 (MPDA); Multiple *Lactobacillus* spp.	Colitis; Inflammation	M1→M2	Redox balance; Flora regulation; Immune homeostasis	ROS scavenging+ Microbiota modulation → Immune rebalancing	[191,193,194]
***DIRECT EFFECTS: PEV Cargo → M1 Activation (Pro-inflammatory/Anti-tumor)***						
M1 Pro-inflammatory	*E. coli*; *E. coli* Nissle 1917; *S. cerevisia*e; *A. muciniphila*	Cancer; Tumor immunity; Infection	→M1	↑ROS, iNOS, TNF-*α*, IL-6; Enhanced phagocytosis; Tumor inhibition	HIF-1/mTORC1/NF-κB activation; TLR recognition; Metabolic shift to glycolysis	[91,139,195,196]
Anti-tumor Immunity	*E. coli* (engineered EVs)	Cancer immunotherapy	→M1	Antigen presentation; T cell recruitment; Tumor regression; Memory immunity	Direct M1 activation → Enhanced adaptive immunity	[65,197]
***TISSUE-SPECIFIC EFFECTS***						
Wound Healing	*L. reuteri*; *L. crispatus*; *L. bulgaricus*; *L. plantarum*	Oral/cutaneous wounds; Diabetic wounds	M1→M2	Accelerated closure; Angiogenesis; ↓Local inflammation	3-HPA/D-lactate → Mitochondrial stabilization → M2; STAT6 signaling	[86,130,153,198]
Bone/Joint	*L. johnsonii*; *L. animalis*; *L. reuteri*	Osteoarthritis; Bone regeneration; Implant infection	M1→M2	↓Synovial inflammation; Cartilage protection; Osseointegration	Glutamine synthetase/mTORC1; SPP1-JAK1-STAT3 pathway	[199‐201]
Neurological	*L. reuteri*; *L. rhamnosus*	Ischemic stroke; Perioperative neurocognitive dysfunction	M1→M2	↓Neuronal apoptosis; Improved brain immune microenvironment	MAPK/NF-κB inhibition in microglia	[202,203]

Note: Direct effects: PEV cargo → Host receptor/pathway → Macrophage polarization. Indirect effects: PEVs → Microbiota modulation → Metabolite production → Macrophage polarization.

Abbreviations: PRR, pattern recognition receptor; TLR, toll-like receptor; SCFA, short-chain fatty acid; NLRP3, NOD-like receptor protein 3; HIF-1, hypoxia-inducible factor; mTORC1, mechanistic target of rapamycin complex 1; NF-κBnuclear factor kappa-B; STAT6, signal transducer and activator of transcription 6; SPP1, secreted phosphoprotein 1; JAK1, Janus kinase 1; MAPK, mitogen-activated protein kinase; STING, stimulator of interferon genes; M1, classically activated macrophage; M2, alternatively activated macrophage; ↑, increased; ↓, decreased.

#### 3.4.1 Anti-inflammatory and tissue-protective ability

Probiotic-derived EVs exert potent immunomodulatory effects primarily through promoting M2 macrophage polarization, enhancing anti-inflammatory cytokine secretion, and facilitating tissue repair across multiple organ systems.^176^^,^^194^ *Lactobacillus-*derived EVs represent the most extensively studied class of immunomodulatory vesicles in intestinal tissues.^194^ Animal studies using mice, chickens, and pigs have consistently demonstrated that these EVs modulate macrophage polarization and reduce inflammatory responses.^204^ Key methodological details across studies include: most mouse studies employed DSS-induced colitis models with C57BL/6 or BALB/c strains; EV doses typically ranged from 10-100 μg protein equivalent per mouse per day; administration routes included oral gavage, intraperitoneal injection, and intravenous delivery; treatment durations spanned 7-21 days^177^^,^^194^^,^^205^; and macrophage targeting was typically assessed by flow cytometry of lamina propria cells and immunofluorescence co-localization. EVs from *Lactobacillus plantarum* induce M2 polarization, promoting IL-10 and TGF-*β* release while suppressing IL-6 and TNF-*α*, thereby facilitating intestinal tissue repair.^194^ Similarly, EVs from *Lactiplantibacillus plantarum* modulate M2 polarization and regulate gut microbiota composition, reducing the required dose of 5-aminosalicylic acid.^100^ EVs from *Lactobacillus johnsonii* enhance gut barrier function,^176^ while miR-146b in Lactococcus lactis EVs shifts macrophages from M1 to M2 phenotype.^95^ In pigs, *Limosilactobacillus* mucosae-derived EVs suppress M1 polarization and inhibit NF-κB and AKT expression, alleviating *E. coli* K88-induced diarrhea.^206^ EVs from *Limosilactobacillus johnsonii* and *Limosilactobacillus* mucosae induce M1-to-M2 phenotypic shift, enhancing tight junction protein expression and suppressing apoptosis-related genes.^207^ In broilers, *Lactobacillus reuteri*-derived EVs suppress pro-inflammatory genes while upregulating IL-10 and TGF-*β.*^183^

Other probiotic-derived EVs exhibit comparable properties. EVs from *Bifidobacterium longum subsp. infantis* downregulate M1 markers (iNOS and CD86), while upregulating M2 markers (iNOS and CD86), attenuating intestinal inflammation.^175^ *Clostridium butyricum* EVs alleviate inflammation by promoting M2 polarization and reshaping gut microbiota.^192^ *Alistipes timonensis* releases outer membrane vesicles containing immunomodulatory sulfonolipids that inhibit pro-inflammatory cytokine expression.^128^

Beyond the intestine, PEVs modulate inflammation in extraintestinal tissues. The mechanisms by which PEVs reach specific tissues (gut, plaques, brain, wounds) involve both passive and active processes. In the gut, EVs traverse the mucus layer and are sampled by M cells overlying Peyer's patches or by CX3CR1+ dendritic cell extensions penetrating the epithelium. Recent studies demonstrated that Akkermansia muciniphila-derived OMVs translocate into Peyer's patches within 2 hours after oral delivery, subsequently migrating to mesenteric lymph nodes and activating B cells and dendritic cells to elicit mucosal IgA responses.^208^ For systemic distribution, EVs must first cross the epithelial barrier via transcytosis or paracellular routes during inflammation to enter the lamina propria, from which they access lymphatics and eventually the systemic circulation. Biodistribution studies using fluorescently labeled EVs demonstrate preferential accumulation in liver, spleen, and sites of inflammation; notably, intravenously administered gut microbiota-derived EVs biodistribute across multiple organs including the brain, liver, stomach, and spleen, while orally administered EVs initially accumulate in the gastrointestinal tract before systemic dissemination.^53^ Under homeostatic conditions, EVs exhibit limited penetration of the blood-brain barrier, but enhanced CNS access occurs during neuroinflammation when barrier integrity is compromised. In atherosclerotic plaques, EVs accumulate through enhanced endothelial permeability characteristic of lesion-prone areas, where they are internalized by plaque-resident macrophages; human atherosclerotic plaques have been observed to contain EVs originating from various cell types including leukocytes, macrophages, erythrocytes, lymphocytes, smooth muscle cells, and platelets.^209^ For wound healing applications, topically applied EVs directly contact wound-bed macrophages, while systemically administered EVs home to wound sites following chemokine gradients (particularly CCL2/MCP-1), promoting the transition of macrophages from pro-inflammatory M1 to anti-inflammatory M2 phenotypes and facilitating tissue repair.^210^

In dermal contexts, EVs induce macrophage-specific cytokine expression,^86^ and lactic acid bacterial EVs elicit IL-10 secretion via formyl peptide receptor 2.^211^ *Lactobacillus bulgaricus* EVs-driven hydrogels induce M2 polarization and enhance skin repair.^198^ In bone tissue, *Lactobacillus*-derived OMVs promote M2 phenotype and tissue repair.^199^^,^^201^ In the central nervous system, *Lactobacillus rhamnosus* OMVs promote M2-type microglial differentiation, alleviating neuroinflammation.^202^ Additionally, *Lactiplantibacillus plantarum* OMVs promote M2 polarization of pulmonary macrophages.^187^

Collectively, these findings demonstrate that PEVs exert immunomodulatory effects by promoting M2 macrophage polarization, characterized by enhanced anti-inflammatory cytokine secretion and suppression of pro-inflammatory mediators. The consistency across diverse probiotic species, animal models, and tissue types underscores the evolutionary conservation of this mechanism. The systemic reach of PEVs—affecting intestinal, dermal, bone, pulmonary, and neural tissues—positions them as promising therapeutic agents for inflammatory conditions affecting multiple organ systems. However, it should be noted that most systemic effects have been demonstrated following parenteral EV administration rather than oral delivery, and whether physiological quantities of endogenously produced EVs achieve sufficient systemic concentrations to replicate these effects remains uncertain.

#### 3.4.2 Activation of host immunity through M1 polarization

While the majority of probiotic-derived vesicles promote anti-inflammatory M2 polarization, certain PEVs activate pro-inflammatory M1 responses, highlighting the context-dependent and strain-specific nature of vesicle-mediated immunomodulation. This functional duality underscores the potential of PEVs to serve as targeted immunostimulatory agents in contexts requiring enhanced antimicrobial defense or tumor immunosurveillance. The OMVs derived from *Escherichia coli* Nissle 1917 (EcN) have been shown to promote macrophage M1 polarization and activate host immune responses. Following phagocytosis of EcN-OMVs by RAW264.7 cells, intracellular production of reactive oxygen species (ROS), TNF-*α*, IL-6, IL-1β, ATP, and nitric oxide (NO) increased significantly, promoting macrophage proliferation, migration, invasion, and M1 polarization.^195^ Engineering strategies have further exploited this immunostimulatory capacity: modified EcN-OMVs have been shown to induce repolarization of M2 macrophages toward the M1 phenotype with concomitant upregulation of inflammatory factors.^212^ Engineered OMVs loaded with immune-responsive gene 1 inhibitor, programmed death ligand 1 nanobody, and poly (lactic-co-glycolic acid) have yielded similar pro-inflammatory effects.^65^^,^^197^ However, strain-specific variations are evident: OMVs produced by *E. coli* O83 (Colinfant Newborn) activate TLR2/4/5 as well as NOD1 and NOD2, inducing splenocytes and bone marrow-derived dendritic cells (BMDCs) to produce IL-10, thereby suppressing inflammatory responses and preventing allergic reactions.^213^ These divergent effects underscore the importance of strain-specific vesicle composition in determining immunological outcomes.

Beyond probiotic strains, vesicles derived from intestinal commensal bacteria can also elicit pro-inflammatory M1 responses. *Segatella copri* OMVs enter macrophages via macropinocytosis and clathrin-dependent mechanisms, inducing a dose-dependent increase in M1-associated surface marker expression in both M0 and M2 macrophages while activating the secretion of numerous pro-inflammatory cytokines in M1 macrophages.^214^ Similarly, fungal EVs can activate host immunity: EVs from Baker's yeast *Saccharomyces cerevisiae*, bearing *β*-D-glucan on their surface, significantly increase the production of pro-inflammatory TNF-*α* and IL-6 in RAW264.7 and DC2.4 cells, along with markedly elevated expression of M1 polarization markers CD40, CD80, and CD86, with TLR2 implicated in immune activation during EVs endocytosis.^91^ Notably, vesicles from some intestinal symbionts may exacerbate inflammatory pathology: EVs from *Bacteroides uniformis* induce M1 macrophage polarization and intensify intestinal inflammatory responses during the weaning period of piglets.^215^

Collectively, these studies provide converging functional evidence that PEVs serve as bidirectional modulators of macrophage polarization, capable of promoting either anti-inflammatory M2 or pro-inflammatory M1 phenotypes depending on vesicle composition, parental strain characteristics, and host immune context. This functional plasticity positions PEVs as versatile immunotherapeutic agents with applications ranging from inflammation resolution to immune activation in cancer or infectious disease settings. However, the molecular determinants underlying strain-specific immunological outcomes, the specific contributions of individual vesicle cargo components, and the mechanisms governing tissue- and context-dependent polarization responses require further investigation. Understanding these factors will be essential for rational design of PEV-based therapeutics with predictable immunomodulatory profiles.

### 3.5 The PEVs modulate macrophage function through distinct molecular mechanisms

**Figure 3.**
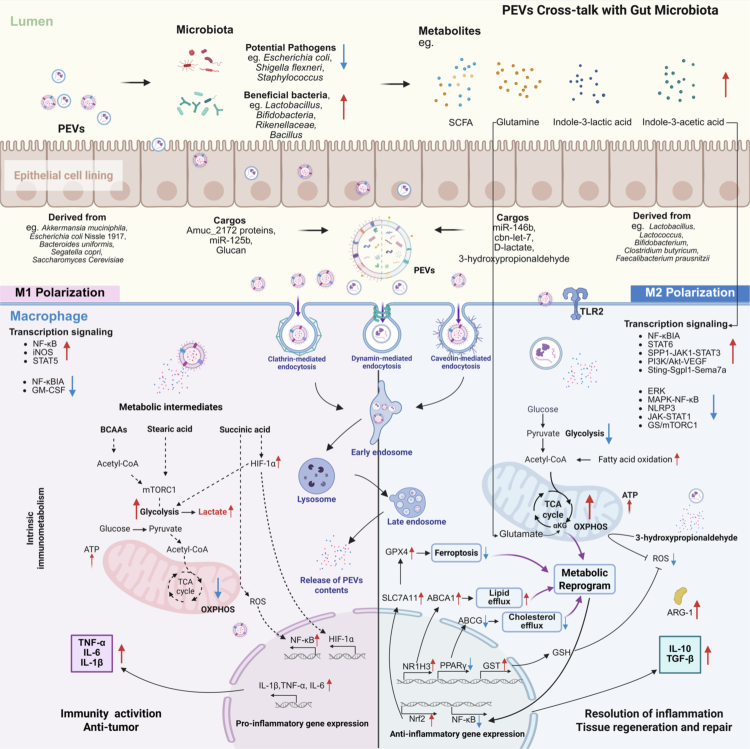
Mechanism of the Probiotics-derived extracellular vesicles (PEVs) reshape macrophage function and metabolic reprogram. PEVs are internalized into macrophages by clathrin-mediated endocytosis, dynamin-mediated endocytosis and caveolin-mediated endocytosis, and exert their immunomodulatory effects through two principal mechanisms: direct engagement of pattern recognition receptor signaling pathways (eg. NF-κB, NF-κBIA, TLR, SPP1-JAK1-STAT3 and NLRP3) and reprogramming of cellular metabolism (such as OXPHOS, glycolysis, FAO, lipid efflux and ferroptosis). PEVs engage in reciprocal cross-talk with the gut microbiota, wherein vesicle-mediated macrophage polarization reshapes microbial community structure, while microbiota-derived metabolites reciprocally reinforce macrophage function metabolic programming. SCFA, short-chain fatty acids; GLUT1, glucose transporter 1; TLR2, Toll-like receptor 2; NF-κB, Nuclear factor kappa-B; iNOS, inducible NO synthase; STAT, Signal transducer and activator of transcription; NF-κBIA, NF-κB Inhibitor Alpha; GM-CSF, Granulocyte-Macrophage Colony Stimulating Factor; HIF-1α, Hypoxia Inducible Factor 1 Subunit Alpha; BCAAS, Branched Chain Amino Acid; OXPHOS, oxidative phosphorylation; ERK, Extracellular regulated protein kinases; MAPK, Mitogen-activated protein kinase; NLRP3, NOD-like receptor thermal protein domain associated protein 3; mTORC1, mechanistic target of rapamycin complex 1; *α*-KG, *α*-Ketoglutaric acid; SPP1, Secreted Phosphoprotein 1; NR1H3, Nuclear Receptor Subfamily 1 Group H Member 3; JAK, Janus kinase; PPAR *γ*, Peroxisome proliferator-activated receptor γ; GST, Glutathione S-transferase; GSH, Glutathione reductase; Nrf2, Nuclear factor erythroid 2-related factor 2; IL-4, interleukin-4; IL-6, Interleukin-6; IL-1β, Interleukin-1β; TGF-*β*, Transforming growth factor-β; ARG-1, Arginase-1; GPX4, Glutathione peroxidase 4; SLC7A11, Solute Carrier Family 7 Member 11; ABCA, ATP-binding cassette transporter A1; ABCG, ATP Binding Cassette Subfamily G Member 1. The figure was created with https://app.biorender.com/.

Macrophages serve as critical mediators of host-microbe interactions, and PEVs have emerged as key modulators of macrophage polarization and function. PEVs exert their immunomodulatory effects through two principal mechanisms: direct engagement of pattern recognition receptor (PRR) signaling pathways and reprogramming of cellular metabolism (Figure 3). These dual mechanisms collectively determine whether macrophages adopt pro-inflammatory (M1) or anti-inflammatory (M2) phenotypes, with profound implications for tissue homeostasis and disease resolution.

#### 3.5.1 Pattern recognition receptor signaling and transcriptional reprogramming

PEVs initiate immunomodulatory responses by engaging host PRRs, particularly TLRs, which trigger downstream transcriptional cascades that dictate macrophage polarization states. *Lactobacillus*-derived EVs predominantly promote anti-inflammatory M2 polarization through multiple convergent signaling pathways. For instance, EVs from *Lactobacillus murinus* activate TLR2 signaling in intestinal macrophages, driving increased IL-10 production and M2-like polarization.^181^ Similarly, *Lactobacillus johnsonii*-derived EVs suppress extracellular signal-regulated kinase (ERK) signaling, thereby inhibiting NLRP3 inflammasome activation in intestinal epithelial cells and mitigating enterotoxigenic *E. coli* K88-induced macrophage dysfunction.^176^

The NF-κB pathway represents a critical node in PEVs-mediated immunomodulation. *Lactobacillus amylovorus*-derived EVs suppress NF-κB activation by upregulating NF-κBIA expression, promoting an immunosuppressive M2 phenotype that facilitates early-life gut development.^185^ Mechanistically distinct yet functionally convergent, EVs from *Clostridium butyricum* restore miR-199a-3p expression, which subsequently interacts with MAPK4 to inhibit both MAPK and NF-κB signaling cascades, thereby attenuating intestinal inflammation.^188^ Similarly, membrane vesicles from *Eubacterium rectale* suppress LPS-induced NLRP3 inflammasome activation, reduce pro-inflammatory cytokine expression (IL-1β, TNF-*α*), and significantly upregulate IL-10, demonstrating potent anti-inflammatory effects in both *in vitro* and *in vivo* models.^186^

Beyond classical TLR-NF-κB axes, PEVs engage alternative signaling pathways to orchestrate macrophage polarization. *Lactobacillus crispatus*-derived EVs containing D-lactate indirectly promote M2 polarization through STAT6 signaling, which blocks HPV16 infection and enhances wound healing processes including cell migration and angiogenesis.^130^ In the context of bone regeneration, *Lactobacillus reuteri*-derived EVs mediate M2 polarization via the SPP1-JAK1-STAT3 signaling axis.^199^ Furthermore, *Pediococcus pentosaceus*-derived membrane vesicles promote M2 polarization and myeloid-derived suppressor cell differentiation in a TLR2-dependent manner, upregulating IL-10, arginase-1, and PD-L1 expression while suppressing activated T cell proliferation. In a dextran sulfate sodium (DSS)-induced acute colitis model, systemic administration of these vesicles prevented colon shortening and preserved crypt architecture.^182^

Notably, not all microbiota-derived EVs promote anti-inflammatory responses. Diarrheal microbiota-derived EVs induce macrophage polarization toward a pro-inflammatory M1 phenotype through miR-125b/NF-κB-mediated mechanisms, increasing TNF-*α* and IL-1β secretion, disrupting tight junction proteins (ZO-1, Occludin), and elevating intestinal permeability, ultimately causing intestinal homeostatic dysfunction.^216^ This finding underscores the context-dependent nature of EV-mediated immunomodulation.

#### 3.5.2 Immunometabolic reprogramming: Bridging bioenergetics and immune function

Emerging evidence demonstrates that PEVs function not merely as immune stimuli but as sophisticated regulators of macrophage metabolic programming, fundamentally altering cellular bioenergetics to influence polarization states. Classical M1 macrophages rely predominantly on aerobic glycolysis to support rapid pro-inflammatory cytokine production, whereas M2 macrophages favor OXPHOS and FAO to sustain tissue repair functions.^217^ PEVs exploit these metabolic dependencies to reprogram macrophage phenotypes through coordinated modulation of multiple bioenergetic pathways.

A primary mechanism by which PEVs modulate macrophage function involves the suppression of bioenergetic pathways coupled with alterations in lipid homeostasis. *Faecalibacterium prausnitzii*-derived EVs exemplify the therapeutic potential of metabolic reprogramming. These vesicles suppress both glycolysis and OXPHOS in macrophages, downregulating peroxisome proliferator-activated receptor gamma (PPARγ) and ATP-binding cassette (ABC) transporters, thereby reducing cholesterol efflux and promoting a fibrotic-protective M2-like phenotype that attenuates colitis-associated fibrosis.^189^ In atherosclerosis models, *Lactobacillus*-derived EVs mitigate plaque formation by activating the NR1H3-ABCA1 axis, enhancing lipid efflux while concurrently suppressing pro-inflammatory cytokine production and promoting M2 polarization.^23^ These findings highlight lipid metabolism as a critical node linking PEV exposure to macrophage functional reprogramming.

In contrast to metabolic suppression strategies, certain PEVs employ an opposing approach by enhancing glycolytic activity to support immune activation. OMVs from *E. coli* Nissle 1917 (EcN) trigger HIF-1, mTORC1, and NF-κB signaling, enhancing glycolysis while inhibiting the TCA cycle. This metabolic shift increases intracellular ROS, pro-inflammatory cytokines (TNF-*α*, IL-6, IL-1β), ATP, and nitric oxide production, thereby augmenting macrophage phagocytosis and proliferation.^195^ This bidirectional metabolic control demonstrates the functional plasticity of PEV-mediated immunomodulation and underscores the context-dependent nature of vesicle-host interactions.

The integration of metabolic reprogramming with signaling pathway modulation represents a sophisticated mechanism through which PEVs achieve sustained phenotypic changes in macrophages. *Lactobacillus*-derived EVs delivered via oxygen-releasing photothermal hydrogels accelerate diabetic wound healing by promoting pro-regenerative macrophage polarization through dual suppression of NF-κB-mediated inflammation and activation of the PI3K/Akt-VEGF signaling axis, demonstrating the integration of transcriptional and metabolic control mechanisms.^198^ Similarly, *Lactobacillus* brevis-derived metabolites attenuate LPS-induced inflammation by inhibiting histone synthesis and activating glutathione metabolism, supporting an anti-inflammatory macrophage phenotype through coordinated epigenetic and metabolic reprogramming.^218^ These examples illustrate how PEVs orchestrate multiple cellular programs simultaneously to achieve comprehensive immunomodulatory effects.

Central to these metabolic reprogramming mechanisms are mitochondria, which emerge as critical checkpoints in PEV-mediated immunometabolic remodeling. *Lactobacillus reuteri*-derived membrane vesicles deliver 3-hydroxypropionaldehyde (3-HPA), a reuterin metabolite that inhibits mitochondrial permeability transition pore (mPTP) opening, stabilizing membrane potential and reducing mitochondrial ROS accumulation. This mitochondrial stabilization suppresses pro-inflammatory activation while promoting M2-like polarization, as evidenced by upregulated CD206 and IL-10 expression.^153^ In diabetic wound models, *Lactobacillus* biofilm derivatives modulate macrophage metabolism by downregulating the JAK/STAT1 signaling axis and promoting oxidative metabolism. Integrated metabolomic and transcriptomic analyzes reveal enhanced glutathione biosynthesis and a shift toward mitochondrial fatty acid oxidation, sustaining a durable M2-like anti-inflammatory phenotype.^219^

Corroborating evidence from microbiota-derived metabolites further supports the centrality of mitochondrial metabolism in macrophage immunomodulation. Gut microbiota-derived glutamine alleviates hepatic ischemia-reperfusion injury by increasing systemic *α*-ketoglutarate, which fuels the TCA cycle and promotes M2 polarization. This metabolic shift enhances OXPHOS, reprogramming macrophage immunometabolism toward anti-inflammatory and tissue repair functions.^220^ Collectively, these studies establish that PEVs deliver complex molecular cargo-including microRNAs, lipids, and metabolites-that coordinately reprogram cellular bioenergetics to modulate inflammatory responses, representing a conserved principle in probiotic-host interactions.

#### 3.5.3 Cargo-mediated modulation: Molecular determinants of immunometabolic regulation

The immunomodulatory capacity of PEVs is fundamentally determined by their molecular cargo, which comprises nucleic acids, lipids, proteins, and metabolites. These bioactive components function as molecular effectors that reprogram host cellular metabolism and immune responses through coordinated engagement of central regulatory pathways, including hypoxia-inducible factor 1-alpha (HIF-1α), mechanistic target of rapamycin (mTOR), and peroxisome proliferator-activated receptors (PPARs). Understanding cargo-specific mechanisms is essential for elucidating how PEVs achieve their diverse immunometabolic effects in recipient macrophages.

Among the diverse molecular constituents, nucleic acids represent a particularly sophisticated class of cargo capable of directly modulating host transcriptional programs. Emerging evidence reveals that bacterial nucleic acids encapsulated within OMVs can traffic to host cell nuclei,^118^ suggesting that probiotic OMVs may modulate gene expression through vesicle-mediated nucleic acid delivery. Several microRNAs, including let-7i,^93^ miR-5119,^94^ miR-4239,^97^ and miR-146b,^95^ have been implicated in PEV-mediated immune regulation. This nuclear trafficking capacity indicates that probiotic vesicles exploit direct transcriptional reprogramming mechanisms to modulate immune and metabolic networks, representing a sophisticated strategy for host-microbe communication.

Beyond nucleic acids, lipid constituents within PEVs serve as critical mediators of immunometabolic regulation through activation of host lipid-sensing pathways. Sphingolipids derived from *Bacteroides fragilis* OMVs, particularly ceramide-1-phosphate (Cer1P), activate host lipid-sensing pathways by interacting with apolipoprotein L9 (APOL9) in dendritic cells.^24^ This interaction initiates the mevalonate pathway and enhances HMG-CoA reductase (HMGCR) activity, ultimately promoting IL-10 secretion. The functional significance of this lipid-induced IL-10 production extends beyond conventional anti-inflammatory effects to encompass comprehensive immunometabolic reprogramming. IL-10 signaling in macrophages suppresses mTORC1 activation through STAT3-induced DDIT4 expression, thereby promoting mitophagy, reducing mitochondrial ROS, and dampening inflammasome activation.^221^ Furthermore, IL-10 enhances oxidative phosphorylation in inflammatory macrophages via mitochondrial arginase-2 (Arg2), reducing succinate accumulation and inhibiting HIF-1α and IL-1β production to favor an anti-inflammatory phenotype.^222^ These findings collectively suggest that PEVs lipid cargo may mimic or amplify endogenous IL-10-mediated metabolic reprogramming, with mitochondrial function and ROS modulation serving as central regulatory nodes.

Complementing the actions of nucleic acid and lipid cargo, metabolites represent a functionally diverse class of PEVs components with context-dependent immunometabolic effects. Short-chain fatty acids (SCFAs) enhance glycolysis and promote M1-like polarization in tumor-associated macrophages, thereby improving anti-tumor immunity in glioma models.^223^ In contrast, the microbial metabolite indole-3-propionic acid (IPA) suppresses glycolysis and M1 polarization via the JNK/MAPK pathway while promoting M2 polarization through PPARγ-dependent enhancement of fatty acid oxidation.^224^ These opposing metabolic effects underscore the remarkable plasticity of microbial metabolites in regulating macrophage immunometabolism and highlight the importance of tissue microenvironment in determining functional outcomes.

Importantly, macrophages can actively recycle phagocytosed bacterial components, including those potentially delivered via EVs, into bioavailable metabolites such as amino acids, glutathione precursors, and itaconate intermediates. These metabolites fuel oxidative phosphorylation and enhance redox balance, thereby promoting anti-inflammatory functions.^225^ Notably, dead bacteria lacking active virulence factors serve as superior metabolic substrates compared to live bacteria, enhancing host antioxidant capacity through glutathione biosynthesis and AMPK-mTORC1 pathway regulation. This metabolic recycling capacity may amplify the immunomodulatory effects of PEVs cargo, creating sustained metabolic reprogramming beyond the immediate vesicle-receptor interaction.

#### 3.5.4 Pathogen-derived EVs: Contrasting paradigms illuminating probiotic mechanisms

Investigations of pathogenic bacterial OMVs provide critical insights into EVs-mediated immunometabolic regulation through inverse mechanisms. OMVs from Gram-negative pathogens such as *E. coli* and *Salmonella* deliver LPS, pore-forming toxins, and proteases directly into macrophages, triggering pyroptosis and apoptosis via caspase-11 and gasdermin D activation.^226^ These pathogenic vesicles induce profound mitochondrial metabolic reprogramming that links immunometabolism to inflammation and cell death signaling, establishing a conceptual framework for understanding how microbial EVs fundamentally alter host cellular bioenergetics.

A defining characteristic of pathogen-derived OMVs is their capacity to promote a metabolic shift from oxidative phosphorylation toward aerobic glycolysis, a hallmark of pro-inflammatory macrophage activation. OMVs from *Porphyromonas gingivalis* induce this glycolytic switch while concurrently activating the NLRP3 inflammasome and inducing pyroptotic cell death through elevated mitochondrial ROS production.^227^ Similarly, *Pseudomonas aeruginosa*-derived EVs promote aerobic glycolysis via the TLR2/4-PI3K/Akt axis, resulting in increased pro-inflammatory cytokine secretion and subsequent tissue damage.^228^ These metabolic alterations represent pathogen exploitation of host immunometabolic circuitry to amplify inflammatory responses and facilitate tissue invasion.

Beyond acute inflammatory responses, pathogenic OMVs demonstrate remarkable versatility in manipulating host transcriptional machinery to achieve diverse immunological outcomes. OMVs from *Fusobacterium nucleatum* enriched with the adhesin FadA activate Rab5a-mediated endocytosis and facilitate nuclear translocation of Y-box binding protein 1 (YB-1), increasing pro-inflammatory cytokine expression including IL-6 and TNF-*α* in macrophages and synovial cells.^229^ Paradoxically, *F. nucleatum* OMVs can also exert tumor-supportive immunosuppressive functions. Vesicles enriched in tryptophanase reprogram tumor-associated macrophages by stimulating local indole production, which induces TDO2-driven kynurenine synthesis. This activates the aryl hydrocarbon receptor (AHR), resulting in upregulation of immunosuppressive mediators including IL-10, TGF-*β*, and SIGLEC15, ultimately promoting tumor immune evasion and immunotherapy resistance.^230^

The mechanistic insights derived from pathogenic OMV studies provide a conceptual framework for understanding probiotic EV functions through inverse logic. While pathogenic vesicles exploit immunometabolic reprogramming to fuel inflammation, cell death, or pathological immune suppression, probiotic EVs appear to exert counterbalancing effects that restore macrophage homeostasis. Specifically, PEVs promote oxidative phosphorylation over glycolysis, stabilize mitochondrial function, reduce pathological ROS production, and favor tissue-reparative M2 polarization. This mechanistic dichotomy suggests that the evolutionary selection pressures shaping commensal versus pathogenic bacteria have generated fundamentally opposing vesicular communication strategies. Understanding these contrasting paradigms illuminates the therapeutic potential of PEVs as natural antagonists of pathogen-induced immunometabolic dysregulation.

#### 3.5.5 Bidirectional cross-talk with the gut microbiota: A systems-level regulatory circuit

PEVs engage in reciprocal cross-talk with the gut microbiota, establishing a self-sustaining regulatory circuit wherein macrophages function as pivotal intermediaries integrating vesicular cargo with microbiota-derived signals. *Lactobacillus plantarum*-derived EVs exemplify this dual functionality by promoting M2 macrophage polarization while enriching beneficial taxa (*Odoribacter*, *Bifidobacteria*, *Muribaculaceae*) and suppressing pathobionts (*Clostridium*, *Staphylococcus*, *Helicobacter*).^100^^,^^191^^,^^194^^,^^231^ Similarly, *Lactobacillus johnsonii*-derived EVs alleviate colitis by enhancing M2-like populations and reshaping microbial communities toward *Rikenellaceae* and *Lachnospiraceae.*^141^ This integration of PEVs effects with microbiota-derived metabolites is further exemplified by *Faecalibacterium prausnitzii* EVs, which simultaneously reprogram gut microbial metabolism, reduce host susceptibility to viral infections, and promote M2b-like macrophage polarization that mitigates colitis-associated fibrosis.^117^ Importantly, the functional consequences of this microbiota restructuring provide mechanistic reinforcement for macrophage phenotypic stability through metabolite-mediated feedback loops.

Microbiota restructuring reinforces macrophage phenotypic stability through metabolite-mediated feedback. *Clostridium butyricum*-derived EVs induce M2 repolarization while restoring SCFA-producing genera, creating metabolic feedback that sustains anti-inflammatory programming through HIF-1α and mitochondrial oxidative modulation.^188,232‐234^ Tryptophan-derived metabolites represent additional critical mediators: indole-3-acetic acid activates AhR signaling to promote M2 polarization,^235^ while indole-3-lactic acid from *Bifidobacterium brevis* facilitates inflammatory-to-steady-state macrophage differentiation via AhR-PI3K/AKT signaling.^236^^,^^237^ Collectively, these findings establish that the gut microbiota functions as an active partner amplifying vesicle-initiated programs through metabolite production. This tripartite vesicle-microbiota-macrophage axis illuminates therapeutic strategies for restoring intestinal homeostasis in inflammatory diseases.

## 4 Therapeutic implications: Translating immunometabolic insights into clinical applications

**Figure 4.**
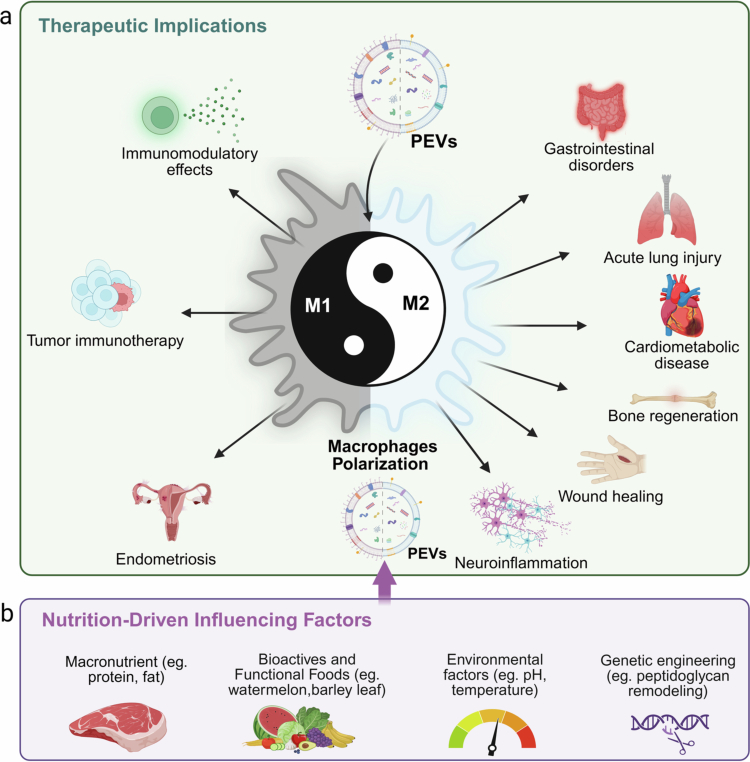
An overview of potential application (a) of the Probiotics-derived extracellular vesicles (PEVs) and influencing factors on its production and functionality (b). The figure was created with https://app.biorender.com/.

The capacity of PEVs to modulate macrophage metabolism—through suppression of glycolysis, enhancement of oxidative phosphorylation, stabilization of mitochondrial function, and coordination with microbiota-derived metabolites—positions these vesicles as promising biotherapeutic agents for inflammatory bowel disease, chronic wound healing, and skeletal tissue regeneration (Figure 4a). However, critical assessment of the current evidence base reveals significant limitations that must be addressed before clinical translation. Human evidence is essentially absent: the vast majority of therapeutic claims derive from mouse studies (primarily C57BL/6 and BALB/c strains with DSS-induced colitis, high-fat diet models, or wound models) with a smaller number of studies in pigs, chickens, and rats. No completed human clinical trials evaluating PEV therapeutics have been published as of this review.

### 4.1 Gastrointestinal disorders: Restoring intestinal homeostasis through macrophage metabolic modulation

Dysregulation of intestinal macrophage metabolism contributes to inflammatory bowel disease pathogenesis.^238^ *Faecalibacterium prausnitzii*-derived EVs alleviate chronic colitis and fibrosis by suppressing both glycolysis and oxidative phosphorylation, downregulating PPARγ and ABC transporters to promote a fibrotic-protective M2-like phenotype.^189^ In the experimental model, male C57BL/6 mice received 2% DSS in drinking water for 7 days to induce acute colitis, followed by 14 days recovery, repeated for 3 cycles, with *F. prausnitzii* EVs (100 μg/mouse/day) administered by oral gavage daily. Macrophage polarization was assessed by flow cytometry using F4/80, CD11b, CD206, and CD86 markers, while biodistribution studies with DiR-labeled EVs demonstrated primary accumulation in the colon and liver.^189^ Similarly, *Lactobacillus plantarum*-derived EVs restore epithelial barrier integrity while promoting M2 polarization,^194^ whereas *Lactobacillus murinus*-derived EVs achieve M2 polarization through TLR2-mediated IL-10 induction.^181^

These bacterial EV-mediated mechanisms are complemented by microbiota-derived metabolites that employ similar immunometabolic strategies. *Alistipes timonensis* releases OMVs containing immunomodulatory sulfonolipids that suppress pro-inflammatory cytokines and alleviate colitis,^128^ while indole-3-propionic acid ameliorates colitis by inhibiting M1 polarization through metabolic reprogramming that suppresses glycolysis and enhances fatty acid oxidation.^224^ Building upon these natural mechanisms, bioengineered PEVs offer additional therapeutic versatility: *Lactobacillus plantarum*-derived EVs encapsulating fucoxanthin scavenge ROS and promote M2 polarization,^191^ whereas hybrid nanovesicles utilizing *E. coli* Nissle 1917 OMV membranes with curcumin-loaded mesoporous polydopamine cores alleviate inflammation through dual ROS scavenging and M2 polarization mechanisms.^193^

### 4.2 Regenerative medicine and tissue repair

In wound healing, *Lactobacillus plantarum*-derived EVs suppress inflammatory cytokines while enhancing IL-10 production in human skin explants.^86^ For diabetic wounds specifically, biofilm-derived postbiotics from *Lactobacillus* accelerate healing by inducing M2 polarization through JAK/STAT1 downregulation,^219^ while *Lactobacillus bulgaricus* EV-loaded oxygen-releasing photothermal hydrogels further enhance M2 polarization and angiogenesis.^198^ Topical application of Lactobacillus-derived EV hydrogels to full-thickness excisional wounds in streptozotocin-induced diabetic mice increased the M2/M1 macrophage ratio from 0.8 to 2.3 by day 7 post-wounding, demonstrating substantial immunomodulatory capacity in the context of impaired diabetic wound repair.^219^

In skeletal regeneration, *Lactobacillus johnsonii*-derived membrane vesicles modulate the glutamine synthetase/mTORC1 pathway to increase M2/M1 ratios and alleviate osteoarthritis.^200^ *Lactobacillus animalis*-derived EVs enhance osteogenic activity while reducing inflammation,^201^ and *Lactobacillus reuteri*-derived EVs promote bone regeneration via the SPP1-JAK1-STAT3 pathway.^199^ Bioengineered EVs loaded with pBMP-2-VEGF and co-encapsulated with IL-4 in GelMA hydrogels successfully accelerate osteoporotic fracture repair through sequential immunometabolic-then-regenerative mechanisms.^239^

### 4.3 Systemic and extraintestinal applications

In cardiometabolic diseases, *Lactobacillus rhamnosus*-derived EVs demonstrate atheroprotective effects through activation of the NR1H3-ABCA1 axis, thereby enhancing cholesterol efflux and promoting M2 macrophage polarization.^23^ Furthermore, they reported that intravenous administration of these EVs (100 μg/mouse, three times weekly for 8 weeks) to high-fat diet-fed ApoE−/− mice resulted in approximately 35% reduction in aortic plaque area, with PKH67-labeled EVs accumulating in atherosclerotic lesions within 24 hours and co-localizing with CD68+ macrophages—suggesting preferential delivery to foam cell-rich regions. Nevertheless, therapeutic outcomes appear highly context-dependent. *Bacteroides fragilis* EVs, while promoting M2 polarization via Sting-Sgpl1-Sema7a signaling under certain conditions, paradoxically exacerbate vascular calcification in type 2 diabetes through stimulator of interferon response cGAMP interactor 1 (Sting)-mediated phosphorylation of myocyte enhancer factor 2D (Mef2d) and subsequent upregulation of serpin family E member 1 (Serpine1).^190^ This context-dependency extends to oncological applications, where *Akkermansia muciniphila* EVs promote M1 macrophage recruitment and reduce tumor burden in prostate cancer models; ^196^ however, tumor-imposed metabolic constraints—including suppression of macrophage glycolysis under hypoxic conditions—introduce additional therapeutic complexity that may limit efficacy in certain microenvironments.^240^

In neuroinflammation, *Lactobacillus rhamnosus*-derived EVs induce M2 microglial differentiation and reduce pro-inflammatory cytokines,^202^ while *Lactobacillus reuteri*-derived EVs prevent neuronal apoptosis following ischemic stroke through ROS clearance and MAPK/NF-κB inhibition.^203^ *Lactiplantibacillus plantarum*-derived EVs mitigate acute lung injury by suppressing ferroptosis while promoting M2 polarization.^187^ Gut microbiota-derived glutamine mediates systemic macrophage reprogramming, conferring hepatic protection through enhanced *α*-ketoglutarate-fueled TCA cycle activity.^220^ These findings establish PEVs as versatile biotherapeutic platforms for redirecting pathological macrophage metabolism. Future efforts should focus on optimizing production scalability, standardizing cargo composition, evaluating long-term safety, and conducting rigorous clinical trials.

### 4.4 Translational challenges and evidence limitations

Critical assessment of the PEV therapeutic literature reveals several significant limitations that must be addressed before clinical translation. Most fundamentally, all therapeutic claims in this field derive exclusively from in vitro cell culture studies and animal models, predominantly mice, with some studies extending to pigs, chickens, and rats. Despite promising preclinical findings, no completed human clinical trials evaluating PEV therapeutics have been published to date.^50^ This represents a critical knowledge gap, as human gut physiology, immune responses, and disease pathophysiology differ substantially from rodent models. The translation of immunometabolic findings from murine macrophages to human monocyte-derived or tissue-resident macrophages remains to be validated. Notably, early clinical efforts with engineered probiotic strains have yielded mixed results; for instance, Synlogic's EcN-based engineered strain SYNB1934 for phenylketonuria achieved a 34% reduction in plasma phenylalanine during its Phase II trial, yet its pivotal Phase III study was terminated in 2024 due to suboptimal efficacy—underscoring the translational gap between preclinical promise and clinical reality.^241^^,^^242^

Beyond the absence of human data, a fundamental mechanistic question remains whether the vesicular delivery system provides advantages over direct administration of active cargo components such as metabolites, proteins, and nucleic acids. Limited comparative studies exist in this regard. Where comparisons have been made, EVs often demonstrate superior efficacy, attributed to their ability to protect cargo from degradation in the harsh gastrointestinal environment, facilitate targeted delivery to specific cell types through surface receptor interactions, enhance cellular uptake via endocytic pathways, and enable co-delivery of synergistic cargo combinations.^243^ The lipid bilayer membrane of EVs provides inherent protection to vulnerable molecular cargoes, including RNA, DNA, and proteins, from enzymatic degradation—a key advantage over free metabolite administration.^244^ However, systematic comparisons controlling for cargo dose, delivery route, and pharmacokinetics remain lacking for most PEV-cargo combinations, leaving this theoretical advantage incompletely substantiated.

Even assuming favorable comparative efficacy, multiple practical hurdles impede PEV clinical translation. Stability remains a primary concern, as EVs are susceptible to degradation by proteases, nucleases, and pH extremes, while lyophilization and cryopreservation can affect their integrity and bioactivity. Current recommendations from the International Society for Extracellular Vesicles (ISEV) advocate cryopreservation at −80 °C, yet even this gold-standard approach may fail to prevent vesicle aggregation, structural compromise, and fragmentation that ultimately impair therapeutic efficacy.^245^ Recent advances in lyophilization using cryoprotectants such as trehalose and sucrose have shown promise in preserving EV structure and bioactivity for extended periods at room temperature, though optimization of shelf-life under various storage conditions remains ongoing.^246^^,^^247^ Closely related to stability concerns is the issue of uptake efficiency, as the fraction of administered EVs that reach target cells in vivo remains unknown. Studies indicate that EVs administered intravenously exhibit a short half-life in the bloodstream, often under 30 minutes, with most vesicles accumulating in the liver and spleen following systemic administration.^248^ Oral bioavailability across the intestinal barrier is similarly poorly characterized, though evidence suggests that orally administered EVs show primary accumulation in the gastrointestinal tract with limited systemic distribution.^249^ Scalability presents another formidable barrier, as current production methods yield only milligram quantities whereas clinical applications may require gram-scale production. In contrast to the low productivity of mammalian EVs, bacterial EVs derived from high-density batch-cultured probiotics possess rapid proliferative capacity, potentially enabling mass production tailored to synthetic biology approaches.^242^ Nevertheless, batch-to-batch variability in cargo composition complicates standardization efforts, and a key challenge lies in the design of industrial processes and optimization of separation and purification operations required for product standardization and quality assessment.^250^ Safety considerations further compound these challenges, encompassing the long-term effects of repeated EV administration, which remain unstudied, along with potential immunogenicity arising from non-self bacterial components. Although probiotic-derived EVs exhibit promising immunomodulatory effects, their safety profile requires careful assessment since, as bacterial products, they may still contain substances capable of causing immunological reactions, particularly lipopolysaccharide (LPS), rendering them potentially pyrogenic if purification is inadequate.^251^ Theoretical concerns also exist regarding horizontal gene transfer via EV-encapsulated DNA, and effects on beneficial microbiota remain incompletely characterized.

Compounding these developmental challenges, pharmacokinetic parameters remain inadequately defined across the field. Route of administration, dosing regimens, and biodistribution have been inconsistently reported across studies. Where biodistribution data exist, typically derived from fluorescent labeling studies, EVs administered orally show primary accumulation in the gastrointestinal tract with variable systemic distribution to the liver, spleen, kidney, pancreas, lung, heart, brain, and colon depending on time of delivery.^249^^,^^252^ Intravenously administered EVs accumulate predominantly in the liver and spleen within one hour of administration.^253^ The relationship between administered dose and tissue concentrations achieved is rarely quantified, making it difficult to establish dose-response relationships or compare findings across studies. Furthermore, methodological inconsistencies in model selection, control design, and effect measurement pose challenges for standardizing outcomes and interpreting results, while understanding biodistribution is critical for assessing therapeutic mechanisms and off-target effects.^50^ Collectively, these limitations underscore the nascent state of PEV therapeutic development and highlight the substantial research agenda that must be addressed before clinical translation can be realized.

## 5 Nutritional regulation of PEVs production and function

The therapeutic potential of PEVs is inextricably linked to their abundance and cargo composition, both profoundly influenced by host nutritional status and dietary patterns. Understanding how dietary interventions influence PEVs production and function is essential for developing nutrition-based therapeutic strategies. Several influencing factors are shown in Figure 4b.

Dietary macronutrient composition exerts pronounced effects on microbiota-derived EVs.^254^ High-protein diets increase EVs production, promote TLR4 activation, and enhance CCL28-mediated IgA secretion through succinate accumulation that stimulates bacterial vesiculation as a stress response.^255^ Conversely, high-fat diets enrich *Pseudomonas panacis*, whose EVs impair insulin signaling and induce glucose intolerance.^256^ These findings suggest dietary interventions could optimize PEVs-based therapeutics through precision nutrition strategies.

Specific dietary bioactives selectively enhance probiotic EVs release and therapeutic efficacy. Watermelon consumption enhances *Lactiplantibacillus plantarum* colonization and EVs production, reinforcing intestinal barrier repair.^257^ Barley leaf supplementation promotes *L. plantarum*-derived EVs release and protects against *Citrobacter rodentium*-induced colitis.^258^ Nuciferine increases *Akkermansia muciniphila* EV secretion, exerting hepatoprotective effects through the miR-23a-3p–SIRT1–NF-κB axis.^259^ Bile acids induce approximately 100-fold increases in EVs release from *Lactobacillus johnsonii.*^52^ Notably, garlic-derived exosome-like nanoparticles “train” *A. muciniphila* to secrete EVs enriched in immunomodulatory proteins including Amuc-1100, reversing high-fat diet-induced type 2 diabetes.^101^

Genetic engineering strategies offer precise control over EV production and cargo composition. Peptidoglycan remodeling drives hypervesiculation in *E. co*li mutants, increasing OMV production over 40-fold.^260^ Engineered *L. plantarum* overexpressing peptidoglycan-modifying enzymes achieves 66-fold increased EVs secretion with enhanced mucosal repair activity.^261^ Manipulation of membrane curvature in *E. coli* DH5α boosts vesiculation nearly 150-fold with demonstrated colitis treatment efficacy.^262^

The convergence of nutritional modulation and genetic engineering presents synergistic therapeutic opportunities. Dietary interventions could prime the gut microenvironment for colonization of engineered probiotic strains, while genetic modifications ensure optimal cargo composition. Future research integrating nutritional manipulation, synthetic biology, and macrophage metabolic profiling will be essential to advance PEVs toward clinical application.

## 6 Conclusions and future perspectives

Probiotic extracellular vesicles have emerged as promising postbiotic effectors bridging microbial signaling and host immunometabolism, offering superior stability and safety compared to live bacteria.^49^^,^^100^ By modulating fundamental bioenergetic pathways—including glycolysis, oxidative phosphorylation, fatty acid oxidation, and mitochondrial dynamics—PEVs redirect macrophage function from pathological inflammation toward tissue repair across inflammatory bowel disease, atherosclerosis, wound healing, neuroinflammation, and cancer. Despite this progress, significant challenges remain: molecular determinants of cargo loading, vesicle targeting, and metabolic reprogramming require systematic elucidation; standardized production protocols, analytical methods, and potency assays must be established; and long-term safety profiles require rigorous preclinical evaluation. Most critically, the translation of promising animal model findings to human patients remains entirely speculative in the absence of clinical trial data.

Converging technological advances create opportunities to accelerate clinical translation. Synthetic biology enables rational design of hypervesiculating strains with optimized cargo through genetic modifications targeting peptidoglycan remodeling and membrane composition,^191^^,^^240^^,^^263^^,^^264^ while smart vesicle platforms responsive to pH, inflammatory cytokines, or hypoxia could enable selective activation within diseased tissues. Defining strain-specific immunometabolic signatures through systematic multi-omics profiling is critical for rational probiotic selection,^204^^,^^265^ with advanced analytical technologies including single-cell sequencing,^266^ spatial omics,^267^ and organoid systems^268^ providing powerful mechanistic tools.

The therapeutic horizon extends to preventive strategies leveraging the diet-microbiota-immunity axis, where precision nutrition approaches modulating endogenous PEVs production could enable non-invasive immunometabolic reprogramming. By integrating mechanistic insights with translational innovation, next-generation PEVs may evolve into precision nanomedicines offering novel therapeutic strategies for chronic inflammatory and metabolic diseases.
